# Biogenic Selenium Nanoparticles from Food-Grade *Pediococcus acidilactici* JD-21: Selenite Bioreduction, Enhanced Probiotic Traits, and Antioxidant Protection

**DOI:** 10.3390/foods15142440

**Published:** 2026-07-09

**Authors:** Shiyue Fan, Jiaxu Li, Xin Zhao, Yi He, Zhiwei Li, Zhangqian Wang, Chao Gao, Ying Ma, Jinquan Li, Xiaoling Chen, Wen Cheng, Xingxing Dong

**Affiliations:** 1 National R&D Center for Se-rich Agricultural Products Processing, Hubei Engineering Research Center for Deep Processing of Green Se-rich Agricultural Products, School of Modern Industry for Selenium Science and Engineering, Wuhan Polytechnic University, Wuhan 430023, China; fanshiyue_0612@163.com (S.F.); 17739012990@163.com (X.Z.); heyi629@126.com (Y.H.); wzqsnu@whpu.edu.cn (Z.W.); gaochao@whpu.edu.cn (C.G.); maying@whpu.edu.cn (Y.M.); chenxl0811@163.com (X.C.); 2 Joint International Research Laboratory of Animal Health and Animal Food Safety, College of Veterinary Medicine, Southwest University, Chongqing 400715, China; tlbzhcq@swu.edu.cn; 3 National Key Laboratory of Agricultural Microbiology, Huazhong Agricultural University, Wuhan 430070, China; lijinquan@mail.hzau.edu.cn; 4 Hengfeng Wanda (Hubei) Pharmaceutical Technology Co., Ltd., Wuhan 430299, China; info@hdimpurity.com

**Keywords:** *Pediococcus acidilactici* JD-21, selenium nanoparticles (SeNPs), selenite reduction, selenium-enriched probiotics, transcriptomic response

## Abstract

Selenite bioreduction by food-grade lactic acid bacteria enables mild production of selenium nanoparticles (SeNPs) together with selenium-enriched biomass. Here, a highly Se(IV)-tolerant isolate from Enshi soil was identified as *Pediococcus acidilactici* JD-21, which efficiently reduced 5 mmol/L Se(IV) and accumulated SeNPs with an average diameter of 46.4 ± 7.7 nm, potentially associated with protein- and polysaccharide-related biomolecules. Selenium enrichment markedly enhanced the antibacterial activity of JD-21 against *Escherichia coli*, *Staphylococcus aureus* and *Salmonella enteritidis* and improved survival in simulated gastric juice, indicating probiotic potential. In a mouse *Streptococcus suis* infection model, oral-gavage SeNPs alleviated infection-associated weight loss, restored antioxidant enzyme activities and reduced liver and spleen lesions. RNA-seq revealed 537 Se(IV)-responsive genes, with up-regulated redox, lipid/exopolysaccharide and transport pathways and down-regulated growth-related functions. These findings demonstrate that JD-21 is a promising food-grade chassis for producing biogenic SeNPs and selenium-enriched probiotics for selenium fortification and foodborne pathogen control.

## 1. Introduction

Selenium (Se) is an essential trace element that plays crucial roles in antioxidant defense, immune regulation, and maintenance of redox homeostasis in humans and animals. However, the biological activity and toxicity of selenium depend largely on its chemical form. Inorganic selenium species such as selenite (SeO_3_^2−^) and selenate (SeO_4_^2−^) are highly toxic at elevated concentrations, whereas organic selenium compounds and nanosized elemental selenium (Se^0^) exhibit high bioavailability and low toxicity [1,2]. Hence, transforming inorganic selenium into safer and more bioavailable forms through green biological approaches has become an important research focus in food chemistry, nutrition, and microbial biotechnology.

Microbial reduction of selenite to Se^0^ or selenium nanoparticles (SeNPs) provides an environmentally friendly strategy for selenium detoxification and utilization. A variety of microorganisms, including bacteria, fungi, and yeasts, are capable of such conversions, but lactic acid bacteria (LAB) have gained increasing attention because of their Generally Recognized as Safe (GRAS) status and broad use in fermented foods [3,4]. Selenium-enriched LAB not only convert toxic inorganic selenium into biologically active forms but also enhance the nutritional and functional quality of food products and probiotic preparations [5,6]. Compared with environmental bacteria such as *Pseudomonas* or *Bacillus*, LAB-based selenium transformation is milder, safer, and more suitable for food applications [7].

Recent studies have revealed that several LAB species can reduce selenite to SeNPs or incorporate selenium into organic molecules. For example, *Lactobacillus casei* ATCC 393 was shown to reduce Se(IV) via a glutathione (GSH)-dependent redox pathway involving glutathione reductase and nitrate reductase [8]. *Lactiplantibacillus plantarum* NML21 demonstrated the capacity to produce uniform spherical SeNPs with high selenium tolerance [9]. Similarly, *Lactobacillus paralimentarius* JZ07 generated red SeNPs (150–300 nm) under Se(IV) stress, with carboxyl and amide groups on cell-surface polymers participating in selenium adsorption and nucleation [10]. Other studies have highlighted the importance of extracellular polymeric substances (EPSs) and surface proteins in stabilizing SeNPs [11,12]. Furthermore, selenium-enriched LAB strains such as *Pediococcus acidilactici* MRS-7 and *Lactiplantibacillus plantarum* S14 have exhibited strong antioxidant and antimicrobial properties and improved survival under gastrointestinal conditions [6,13]. At the systems level, multi-omics analyses have begun to unravel selenium-responsive regulatory networks in LAB, indicating the involvement of redox-active enzymes, thiol metabolism, and stress-related signaling pathways [14,15]. Moreover, animal studies have shown that SeNPs can enhance antioxidant status, modulate immune or inflammatory responses, and protect against oxidative stress-related tissue injury, supporting the need for in vivo evaluation of their functional activity [16,17,18].

Although these studies have provided valuable insights into the selenium conversion ability of LAB, the current understanding of how LAB adapt to and tolerate high concentrations of selenite remains limited. Most reported strains can only withstand relatively low Se levels, and few studies have systematically examined the coordinated molecular responses underlying selenium reduction and tolerance. Additionally, the relationship between intracellular redox regulation, SeNP formation, and probiotic functionality is still unclear. Addressing these questions is essential to better harnessing LAB as efficient and safe microbial platforms for selenium biofortification and functional food development.

In this context, we isolated a highly selenium-tolerant strain, *Pediococcus acidilactici* JD-21, capable of reducing high concentrations of sodium selenite to red SeNPs. This study systematically investigated its selenium transformation characteristics, nanoparticle morphology, probiotic properties, and safety. Furthermore, transcriptomic analysis was performed to explore the molecular responses to selenium stress. The findings of this work provide new evidence for understanding selenium metabolism and adaptive mechanisms in LAB and contribute to the rational application of selenium-enriched probiotics in food and health industries.

## 2. Materials and Methods

### 2.1. Isolation and Identification of Selenite-Reducing Strains

All strains were originally isolated from seleniferous soils sampled from Enshi County, Hubei Province, China, which is reputed as “The World Capital of Selenium”. Briefly, 10 g of soil sample was suspended in 90 mL of phosphate-buffered saline (PBS). It was subjected to shaking at 200 rpm for 30 min at 30 °C and then kept under static conditions for 5 min. The upper suspension was centrifuged at 5000 rpm for 15 min, and the pellets were suspended in 10 mL of PBS. Then 1 mL of suspension was inoculated in 50 mL of De Man, Rogosa and Sharpe medium (MRS medium; Qingdao High-tech Industrial Park Haibo Biotechnology Co., Ltd., Qingdao, China) and incubated for 6 h at 37 °C under shaking at 180 rpm. The enrichment culture was serially diluted and spread on MRS agar and incubated for 48 h at 37 °C. All strains were further purified by streak plating and then were preliminarily identified and numbered based on their morphological characteristics. A 16S rDNA sequencing analysis was performed to identify all isolates and was conducted by Sangon Biotechnology Co., Ltd. (Shanghai, China). The sequence was compared with all the available sequences in the GenBank database with the Basic Local Alignment Search Tool (BLAST) program (http://blast.ncbi.nlm.nih.gov, accessed on 16 March 2022). Multiple-sequence alignment of 16S rRNA gene sequences was performed using ClustalW implemented in MEGA-X (version 10.0.5). A phylogenetic tree was constructed using the neighbor-joining (NJ) method with 1000 bootstrap replications to assess the reliability of the branches.

### 2.2. Growth and Biotransformation of JD-21 Under Se(IV) Stress

The isolates were inoculated in MRS medium containing concentrations of selenite (0, 5, 10, 25, 50, and 100 mmol/L) and incubated for 36 h at 37 °C. The maximum concentration at which red SeNPs were produced was identified as the selenite tolerance limit. To determine the effect of selenium on the growth of the strain, the optical density (OD) of the strain’s fermentation broth was measured every three hours at a wavelength of 600 nm using a UV spectrophotometer. Each group repeated three times.

To determine the Se(IV) reduction efficiency of strain JD-21, the culture was incubated in MRS medium supplemented with 5 mmol/L Na_2_SeO_3_ at 37 °C and 180 rpm for 72 h. Samples were collected at 12 h intervals and centrifuged at 12,000 rpm for 20 min, and the supernatant was digested with HNO_3_ for selenium determination using inductively coupled plasma–mass spectrometry (ICP-MS; Agilent 1290II-8900, Agilent Technologies Inc., Santa Clara, CA, USA) [19,20].

The residual Se(IV) concentration in the supernatant was calculated as follows:

$$C_{t}=\frac{(P-P_{0})\times V\times f}{v}$$

where: C_t_: residual Se(IV) concentration in culture supernatant (μg/mL);

P: Se concentration in sample solution measured by ICP-MS (μg/mL);
P_0_: Se concentration in blank control (μg/mL);
V: volume of digestion solution (mL);
v: volume of original sample used for digestion (mL);
f: dilution factor.

The Se(IV) reduction rate was calculated using the following equation:

$$Reduction\;rate\left(\%\right)=(1-\frac{C_{t}}{C_{0}})\times 100$$

where C_0_: initial Se(IV) concentration in the medium (μg/mL).

### 2.3. Subcellular Localization and Structural Characterization of Selenium Nanoparticle Biosynthesis

To compare the Se(IV)-reducing activity associated with different cellular fractions, an assay was conducted according to Huang et al. [21] with minor modifications. Subcellular fractions, including periplasmic proteins, cytoplasmic extracts, cell membrane components, extracellular polysaccharides, and culture supernatants, were prepared and used as potential SeNP-producing sources. A reaction mixture consisting of 100 µL of each fraction, 88 µL of McIlvaine buffer, 10 µL of Na_2_SeO_3_ solution, and 2 µL of NADH was dispensed into 96-well plates and incubated at 37 °C for 48 h. Control reactions without exogenous NADH/NADPH, without cellular fractions/protein sources, and without Se(IV) were included to evaluate endogenous reducing activity, spontaneous reduction, and background interference. The appearance of red coloration was used as a preliminary indication of elemental selenium [Se(0)] formation. Because this assay was performed using isolated cellular fractions under in vitro reaction conditions, the results were interpreted as fraction-associated Se(IV)-reducing activity rather than definitive evidence of in situ subcellular localization of SeNP biosynthesis.

To further validate the subcellular localization results and characterize the morphology and surface functional groups of the biosynthesized SeNPs, scanning electron microscopy (SEM) and Fourier Transform Infrared Spectroscopy (FT-IR) analyses were subsequently performed. The strain was cultured in MRS supplemented with 5 mmol/L Na_2_SeO_3_ at 37 °C and 180 rpm for 48 h, followed by centrifugation at 12,000 rpm for 10 min to collect the bacterial cells. A part of the precipitated cells was disrupted using an ultra-high-pressure, low-temperature cell disruptor (JN-MINIPRO, Guangzhou Juneng Nano & Bio Technology Co., Ltd., Guangzhou, China) at 4 °C under 1500 MPa. The resulting suspension was centrifuged again (12,000 rpm, 10 min), and the pellet (non-disrupted and disrupted) was fixed with 2.5% glutaraldehyde at 4 °C overnight. For FT-IR analysis, another portion of the bacterial culture was freeze-dried, ground, and homogenized with potassium bromide (KBr) to form transparent pellets. The samples were then subjected to FT-IR spectral scanning in the range of 400–4000 cm^−1^ [21].

### 2.4. Determination of Total Selenium and Selenium Speciation

Strain JD-21 was inoculated into MRS supplemented with 5 mmol/L Na_2_SeO_3_ and incubated at 37 °C for 24 h and 48 h. The cultured cells were harvested by centrifugation (8000 rpm, 10 min), washed three times with sterile saline, and freeze-dried under vacuum. For total selenium determination, 0.05 g of lyophilized biomass was digested with HNO_3_, diluted to an appropriate volume, and quantified using liquid chromatography–atomic fluorescence spectrometry (LC-AFS8530, Haiguang Instrument Co., Ltd., Beijing, China) [22].

For selenium speciation analysis, samples were collected at 48 h under the same cultivation conditions described above. A total of 0.1 g of the dried sample was resuspended in sterile water and disrupted using an ultra-high-pressure, low-temperature cell disruptor. Subsequently, 2 mL of the lysate was mixed with 1 mL of enzymatic hydrolysis solution containing 2 mg of protease E and incubated at 37 °C for 24 h, followed by ultrasonication for 30 min. After centrifugation (8000 rpm, 10 min), the supernatant was collected and filtered through a 0.22 μm nylon membrane. Selenium species in the extract were identified and quantified using high-performance liquid chromatography coupled with inductively coupled plasma–mass spectrometry (HPLC-ICP-MS). The Se species were identified by comparing their retention times with those of selenium standards, including SeCys_2_, MeSeCys, Se(IV), SeMet, and Se(VI). This selenium speciation procedure was adapted from previously reported HPLC-ICP-MS-based methods for selenium-enriched microbial and lactic acid bacterial samples, in which enzymatic hydrolysis and cell disruption were used to release intracellular and protein-associated selenium species prior to chromatographic separation and ICP-MS detection [23,24]. The working parameters and elution gradients are listed in Supplementary Materials Table S1.

### 2.5. Antimicrobial Activity of Selenium-Enriched JD-21 Cultures

Strain JD-21 was inoculated into MRS with or without 5 mmol/L Na_2_SeO_3_ and incubated at 37 °C with shaking at 180 rpm for 24 h. Pathogenic strains (*Escherichia coli* ATCC 18683, *Staphylococcus aureus* ATCC 6538 and *Salmonella enteritidis* ATCC 13076) were harvested from fresh agar plates, resuspended in PBS, and adjusted to an optical density at 600 nm (OD_600_) of 0.5. For the agar diffusion assay, 15 mL of LB agar was poured into a sterile Petri dish to form the base layer. After solidification, three sterile Oxford cups were placed on the agar surface. Subsequently, 5 mL of LB agar containing 1% (*v*/*v*) of the standardized pathogen suspension was poured onto the base layer. After solidification, Oxford cups were pulled out. One well was filled with 200 μL of sterile MRS medium (negative control), and the remaining wells were filled with 200 μL of JD-21 culture without selenium or selenium-enriched JD-21 culture. Plates were incubated at 37 °C for 24 h, and the inhibition zones around each well were then recorded [22,25].

### 2.6. Survival of Selenium-Enriched JD-21 in Simulated Gastric Juice

Pepsin was dissolved in sterile PBS adjusted to pH 3.0 to prepare simulated gastric juice containing 3 g/L pepsin, and the solution was sterilized by filtration through a 0.22 µm membrane filter. Strain JD-21 was cultured in MRS at 37 °C for 12 h. Then, 1 mL of the JD-21 culture was inoculated into 9 mL of the simulated gastric juice and incubated at 37 °C under shaking at 90 rpm for 3 h. Viable cell counts at 0 h and 3 h were determined by plate counting on MRS agar after appropriate serial dilutions. The survival rate was calculated according to the following equation [21,26]:

$$Survival\;rate(\%)=\frac{N_{3h}}{N_{0h}}\times 100$$

where $N_{0h}$ and $N_{3h}$ epresent the viable counts (CFU/mL) of JD-21 at 0 h and 3 h, respectively.

### 2.7. In Vivo Anti-Infective Activity of SeNPs Against Streptococcus suis

Male mice (*n* = 8 per group) were randomly assigned to four groups: (1) control group, (2) SeNP group, (3) *S. suis* infection group, and (4) SeNP + *S. suis* treatment group. Randomization was performed after acclimatization to minimize allocation bias. Investigators responsible for biochemical assays and histopathological evaluation were blinded to the group allocation whenever applicable. Mice in the SeNP group and SeNP + *S. suis* treatment group received SeNPs by oral gavage at a dose of 1.0 mg SeNPs/kg body weight once daily for 15 consecutive days, while the control and *S. suis* infection group were administered with an equal volume of water. The dose of 1.0 mg/kg was selected based on preliminary dose–response observations and previous reports showing that this dose range of SeNPs is well tolerated and biologically effective in mice. On day 8, each mouse in the *S. suis* infection group and SeNP + *S. suis* treatment group was inoculated by intraperitoneal injection with 0.2 mL of a *Streptococcus suis* suspension in PBS (6 × 10^9^ CFU/mL). All animals were maintained under controlled environmental conditions (25 ± 2 °C, 12 h light/dark cycle) with free access to standard chow and water. Feed intake was recorded daily at the same time, and clinical signs, including responsiveness, general appearance, mental state, and coat condition, were monitored throughout the experiment. Body weight was measured every two days [27]. Humane endpoints were predefined before the experiment. Mice were humanely euthanized if they showed severe or persistent distress, including body weight loss exceeding 20%, inability to access food or water, persistent recumbency, severe lethargy, labored breathing, hypothermia, neurological symptoms, or moribund condition. Animals were monitored at least once daily, and more frequently after bacterial challenge. At the end of the experiment, blood samples were collected, and the mice were euthanized. Serum, liver, and spleen tissue homogenates were prepared, and the activities of superoxide dismutase (SOD), total antioxidant capacity (T-AOC), and glutathione peroxidase (GSH-Px) were determined using commercial ELISA kits according to the manufacturers’ instructions. For histopathological analysis, liver and spleen tissues were fixed in 4% (*w*/*v*) paraformaldehyde, dehydrated through a graded ethanol series, embedded in paraffin, sectioned, and stained with hematoxylin and eosin (H&E) for microscopic examination [5]. 

The present study was conducted in strict accordance with the ethical standards for animal experimentation. The animal study protocol was reviewed and approved by the Animal Ethics Committee of Wuhan Myhalic Biotechnology Co., Ltd. (protocol code: HLK 20230921-004; date of approval: 21 September 2023). Throughout the present research study, we upheld the highest standards of animal care and welfare, ensuring that all procedures were consistent with national and international guidelines on humane treatment of animals. This included the implementation of measures to alleviate pain and distress, such as appropriate use of anesthesia and analgesia, as well as the application of the 3Rs principle—Replacement, Reduction, and Refinement—to optimize scientific rigor while minimizing animal usage.

### 2.8. Comparative Transcriptomic Analysis of JD-21 by RNA-Seq

JD-21 was cultivated in MRS supplemented with or without 5 mmol/L Na_2_SeO_3_, and cells were harvested after 7 h of incubation for comparative transcriptomic analysis. Total RNA was extracted from each sample using a Total RNA Isolation Kit (Sangon Biotech, Shanghai, China) according to the manufacturer’s instructions. RNA concentration and purity were assessed with a NanoDrop spectrophotometer (Thermo Fisher Scientific, Waltham, MA, USA). High-quality RNA samples were used for library construction, and sequencing was performed on an Illumina HiSeq platform (Novogene, Beijing, China). Raw sequencing reads were subjected to quality control and filtering with SOAPnuke to remove adaptor sequences, low-quality reads, and reads containing ambiguous bases, yielding high-quality clean reads. Clean reads were then aligned to the JD-21 reference genome using SOAP2 (v2.2.1), and gene-level read counts were obtained for subsequent analysis.

FPKM values were calculated to evaluate overall expression distribution and sample comparability, and density distribution plots were used to assess global expression consistency across samples. For differential expression analysis, expected count data were used as input. Genes with low expression levels were filtered by removing those with counts per million (CPM) < 1 in at least two samples. Raw count data were normalized using the trimmed mean of M-values (TMM) method implemented in the edgeR package in R software (v4.5.1). Dispersion was estimated under a negative binomial generalized linear model (GLM) framework, including common, trended, and tagwise dispersion estimates. Differential expression between selenium-supplemented and control cultures was assessed using a likelihood ratio test. Genes with a false discovery rate (FDR) < 0.05 and an absolute log_2_ fold change (|log_2_FC|) ≥ 1 were considered significantly differentially expressed. To visualize differential expression patterns, MA plots and volcano plots were generated based on log_2_ fold change and FDR values. Functional annotation and enrichment analyses of DEGs were performed based on the Gene Ontology (GO) and Kyoto Encyclopedia of Genes and Genomes (KEGG) databases [15,28].

### 2.9. Statistical Analysis

Statistical analyses were performed using GraphPad Prism v8.3.0 software (GraphPad Software, San Diego, CA, USA). Data are presented as the mean ± standard error of the mean (SEM). The normality of data distribution and homogeneity of variance were assessed before statistical analysis. For comparisons between two groups, an unpaired two-tailed Student’s *t*-test was used when the data were normally distributed and showed equal variances. For comparisons among multiple groups, one-way analysis of variance (ANOVA) followed by an appropriate post hoc test was performed. When the data did not meet the assumptions of normality or equal variance, non-parametric tests, including the Mann–Whitney U test for two-group comparisons and the Kruskal–Wallis test for multiple-group comparisons, were used. All statistical tests were two-tailed, and *p* < 0.05 was considered statistically significant.

## 3. Results and Discussion

### 3.1. Isolation and Identification of the Se(IV)-Reducing Strain JD-21

**Figure 1:**
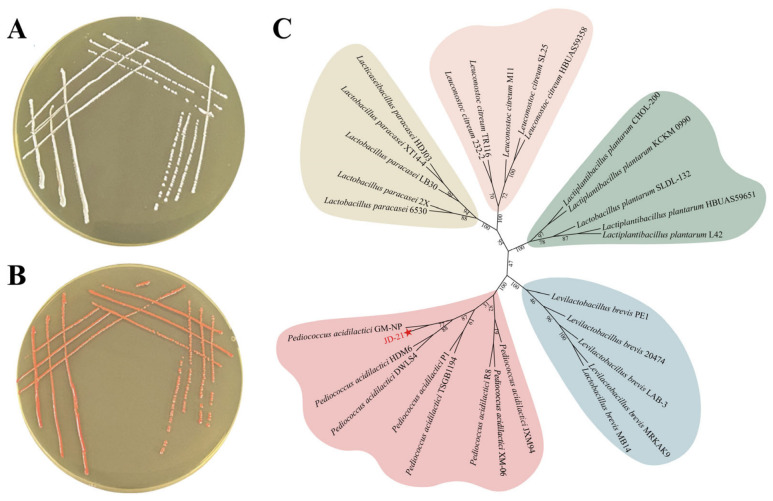
Colony morphology of strain JD-21 on (**A**) MRS agar and (**B**) MRS agar supplemented with 5 mmol/L Na_2_SeO_3_, showing selenite-induced red pigmentation. The colony morphology image was taken from a standard 90 mm Petri dish. (**C**) Phylogenetic tree of JD-21 based on 16S rRNA gene sequences. The strain JD-21 in the present study are highlight in red and labeled with star.

A total of 70 isolates were obtained from soil samples collected in Enshi. Among these, one isolate formed milky-white, round colonies with a smooth and moist surface on MRS agar (Figure 1A). When grown on MRS plates supplemented 5 mmol/L Na_2_SeO_3_, the colony size showed no obvious change, whereas the colony color gradually shifted from milky white to red (Figure 1B). This selenite-induced red pigmentation suggested the in situ formation of elemental selenium nanoparticles (SeNPs) [29]. This strain was designated JD-21. The 16S rRNA gene sequence analysis showed that JD-21 shared 99.66% similarity with *Pediococcus acidilactici*. Phylogenetic analysis based on the 16S rRNA gene clustered JD-21 together with *P. acidilactici* strains (Figure 1C), supporting its identification as *P. acidilactici*. This strain has been deposited in the China Center for Type Culture Collection (CCTCC) under accession number CCTCC M 20241452.

### 3.2. Se(IV) Tolerance, Growth Response, and Selenite Bioreduction Capacity of JD-21

**Figure 2:**
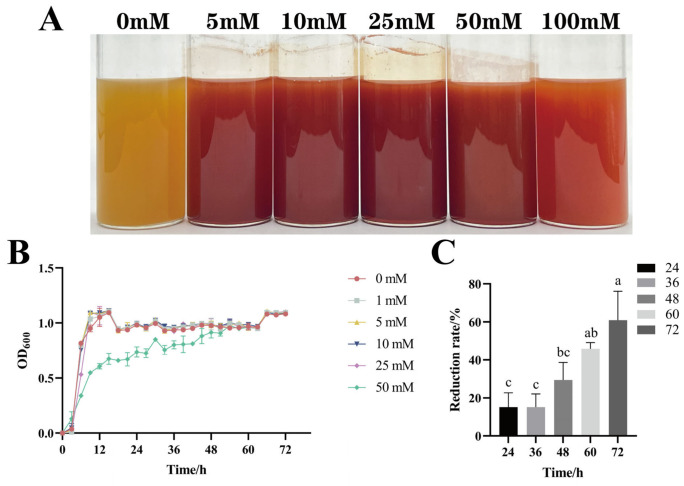
Effect of Se(IV) on the growth and Se(IV)-reducing capacity of *Pediococcus acidilactici* JD-21. (**A**) Effect of different Na_2_SeO_3_ concentrations (5, 10, 25, 50, and 100 mmol/L) on the growth of JD-21 in MRS medium. (**B**) Growth curves of JD-21 in MRS supplemented with 0, 1, 5, 10, 25, and 50 mmol/L Na_2_SeO_3_. (**C**) Time-dependent reduction of 5 mmol/L Na_2_SeO_3_ by JD-21 over 72 h (a–c).

Among the 70 isolates obtained from Enshi soil, 22 strains exhibited Se(IV) tolerance at Na_2_SeO_3_ concentrations higher than 25 mmol/L. JD-21 showed strong tolerance, being able to grow in the presence of up to 100 mmol/L Na_2_SeO_3_, and was therefore selected for further investigation as a highly Se(IV)-tolerant strain of *Pediococcus acidilactici*. When JD-21 was cultivated on MRS agar containing 5 mmol/L Na_2_SeO_3_, dark-red precipitates appeared, and the cells became pigmented (Figure 2A), indicating that the strain reduced Se(IV) to red elemental selenium [10].

As shown in Figure 2B, JD-21 reached its maximal growth at approximately 14 h in MRS without Na_2_SeO_3_. At low Na_2_SeO_3_ concentrations, the growth of JD-21 was not markedly affected compared with the control. In contrast, 25 mmol/L Na_2_SeO_3_ caused a slight delay and reduction in growth, and 50 mmol/L Na_2_SeO_3_ markedly inhibited cell proliferation, indicating that high Se(IV) levels exert an inhibitory effect on JD-21. Considering both Se(IV) tolerance and growth performance, 5 mmol/L Na_2_SeO_3_ was selected as the working concentration for subsequent experiments. The time course of Se(IV) reduction by JD-21 is shown in Figure 2C. The proportion of Se(IV) removed from the medium increased progressively with incubation time, reaching a maximum at 72 h, when 60.85% of the initial selenite had been converted into other selenium species. Previously reported probiotic strains typically show Se(IV) reduction rates of approximately 20–70% at relatively low Na_2_SeO_3_ concentrations, most often below 3 mmol/L [3]. These results indicate that JD-21 combines a relatively high Se(IV) reduction capacity with Se(IV) tolerance far exceeding 3 mmol/L, highlighting its potential as a Se(IV)-resistant, SeNP-producing probiotic candidate.

### 3.3. Selenium Accumulation and Intracellular Speciation in JD-21

**Figure 3:**
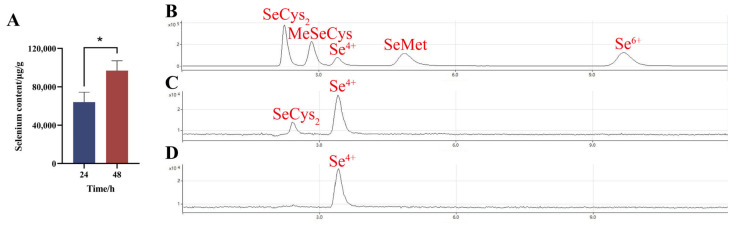
Selenium speciation and accumulation in *Pediococcus acidilactici* JD-21. (**A**) Total selenium content in JD-21 biomass after 24 h and 48 h of cultivation with 5 mmol/L Na_2_SeO_3_. Single asterisk (*) indicates significant difference between the two groups (*p* < 0.05). (**B**) Selenium standard solution (80 µg/mL) containing SeCys_2_, MeSeCys, Se(IV) as selenite SeMet and Se(VI) as selenate. (**C**) Selenium speciation in JD-21 cells cultivated in MRS medium with 1 mmol/L Na_2_SeO_3_. The detected SeCys_2_ and Se(IV) contents were 0.1362 ± 0.0075 μg/g and 0.5479 ± 0.0313 μg/g dry weight, respectively. (**D**) Selenium speciation in JD-21 cells cultivated in MRS medium with 5 mmol/L Na_2_SeO_3_. The chromatographic profile was dominated by Se(IV), with a detected content of 2.3235 μg/g dry weight, while SeCys_2_ remained at a low level of approximately 0.1500 μg/g dry weight. Peaks are labeled according to the corresponding selenium species identified using selenium standards.

The selenium enrichment capacity of JD-21 during fermentation was first evaluated by measuring the total selenium content in *Pediococcus acidilactici* biomass. As shown in Figure 3A, in the 5 mmol/L Na_2_SeO_3_ treatment group, the selenium content of JD-21 pellets reached 64,177.33 ± 7523.56 µg/g (≈64.18 mg/g, dry weight) at 24 h and further increased to 96,812.00 ± 7261.33 µg/g (≈96.81 mg/g) at 48 h, indicating highly efficient selenium accumulation under these conditions. Previous studies have reported that *Lactiplantibacillus plantarum* 6076 and *Lactococcus lactis* 23185 accumulated 4.88 ± 0.39 mg/g and 3.68 ± 0.23 mg/g selenium, respectively, when cultivated with 10 µg/mL Se [25]. These comparisons highlight JD-21 as a promising strain for the preparation of selenium-enriched probiotic biomass.

To further elucidate the intracellular forms of selenium, selenium speciation analysis was performed by HPLC-ICP-MS. As shown in Figure 3B, chromatographic separation of selenium standards resolved five species: selenocystine (SeCys_2_; retention time 2.3 min), methylselenocysteine (MeSeCys; 2.8 min), Se(IV) as selenite (Se^4+^; 3.3 min), selenomethionine (SeMet; 4.8 min), and Se(VI) as selenate (Se^6+^; 9.8 min). In JD-21 cells cultivated with 1 mmol/L Na_2_SeO_3_ (Figure 3C), SeCys_2_ and residual Se(IV) were the main detectable species in the soluble fraction. The SeCys_2_ peak (around 2.3–2.5 min) corresponded to 0.1362 ± 0.0075 µg/g (dry weight), whereas the Se^4+^ peak at 3.3 min accounted for 0.5479 ± 0.0313 µg/g. When the Na_2_SeO_3_ concentration was increased to 5 mmol/L, the chromatographic profile of JD-21 (Figure 3D) was dominated by the Se^4+^ peak at 3.3 min, with a concentration of 2.3235 µg/g, while the SeCys_2_ signal remained at a low level (approximately 0.1500 µg/g) without a marked increase. Given that the most frequently reported organic selenium species in lactic acid bacteria are SeMet and SeCys_2_ [30], these results suggest that under higher selenite stress a smaller fraction of Se(IV) is incorporated into low-molecular-weight organoselenium forms, and a larger proportion is likely reduced to insoluble or nanoparticulate selenium that is not recovered in the soluble fraction analyzed by HPLC-ICP-MS.

### 3.4. Localization and Characterization of Selenium Nanoparticles

**Figure 4:**
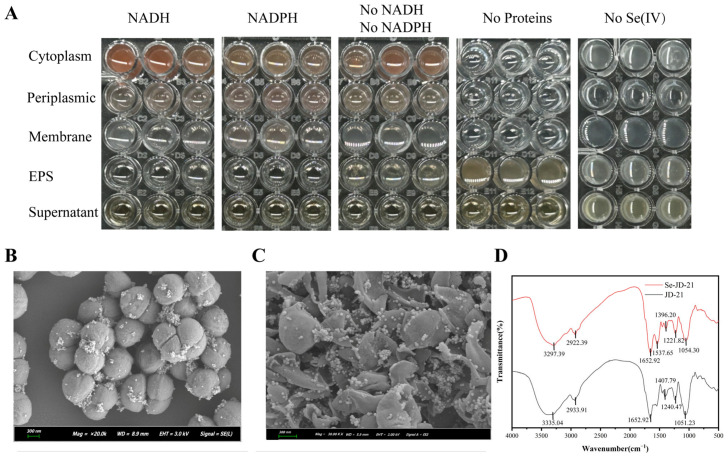
Localization and characterization of selenium nanoparticles produced by *Pediococcus acidilactici* JD-21. (**A**) Se(IV)-reducing activity associated with different cellular fractions of JD-21 under in vitro reaction conditions. Cytoplasmic, periplasmic, membrane-associated, EPS, and supernatant fractions were incubated with Se(IV) in the presence of NADH or NADPH. Control reactions without exogenous NADH/NADPH, without cellular fractions/protein sources, and without Se(IV) were included. Red coloration indicates the formation of elemental selenium [Se(0)]. (**B**) TEM image of intact JD-21 cells grown with selenite, showing selenium nanoparticles on the cell surface. (**C**) TEM image of JD-21 after cell disruption, revealing selenium nanoparticles associated with both the inner and outer cell surfaces. (**D**) Fourier transform infrared (FT-IR) spectra of JD-21 cultures grown in MRS broth with or without 5 mmol/L Na_2_SeO_3_.

To compare the Se(IV)-reducing activity associated with different cellular fractions of JD-21, cytoplasmic, periplasmic, membrane-associated, EPS, and supernatant fractions were incubated with Se(IV) in the presence or absence of NADH/NADPH. The cytoplasmic fraction showed the most obvious red coloration, indicating stronger Se(IV)-reducing activity under the tested in vitro conditions, whereas the periplasmic, membrane-associated, EPS, and supernatant fractions showed weak or limited color development (Figure 4A). The control reactions without Se(IV) did not show red coloration, suggesting that the observed color change was associated with Se(IV) reduction. In addition, reactions without cellular fractions/protein sources showed no obvious red product formation, excluding substantial spontaneous reduction under the assay conditions. These observations are consistent with previous findings that multiple enzymes located in different cellular compartments can participate in bacterial selenite reduction [31], and agree with reports of SeO_3_^2−^-reducing activity in the cytoplasmic protein fraction of Providencia rettgeri HF16 [32].

The localization and morphology of JD-21-derived selenium nanoparticles were further examined by TEM. In cells grown with selenite, numerous electron-dense nanoparticles were observed on or near the cell surface (Figure 4B). The cells remained plump and smooth with clear boundaries, suggesting that selenium exposure did not cause obvious morphological damage to JD-21 under the tested conditions. After cell disruption, a large number of selenium nanoparticles were also visible in cell-associated regions and on exposed cellular fragments (Figure 4C). Together with the in vitro subcellular fraction assay, these observations suggest that Se(IV)-reducing activity in JD-21 is mainly associated with the cytoplasmic fraction, although the precise intracellular formation and translocation process of SeNPs requires further validation.

The SeNPs synthesized by JD-21 were predominantly spherical. Particle size analysis showed that the average diameters of SeNPs in intact-cell and disrupted-cell images were 45.5 ± 6.5 nm (*n* = 67) and 47.2 ± 8.7 nm (*n* = 68), respectively. The combined analysis of 135 particles showed an average diameter of 46.4 ± 7.7 nm, with most particles distributed within approximately 35–55 nm (Figure S2). These results indicate that JD-21-derived SeNPs were nanoscale particles with a relatively narrow size distribution. Selenium nanoparticles with diameters below 200 nm are generally reported to exhibit enhanced biological effects, and smaller SeNPs often show higher biological activity. Wang et al. [9] further demonstrated that the antiproliferative activity of SeNPs against cancer cells is inversely related to particle size.

The functional groups associated with JD-21-derived SeNP-containing biomass were analyzed by FT-IR by comparing samples grown in selenium-free and selenium-containing media (Figure 4D). The broad band at 3292 cm^−1^ was attributed to O–H and N–H stretching vibrations, indicating the presence of hydroxyl- and amino-containing biomolecules, such as polysaccharides, proteins, or peptides. The band at 2925 cm^−1^ corresponded to C–H stretching vibrations, which are typically associated with aliphatic chains in lipids and protein side chains. Characteristic bands at 1657 and 1537 cm^−1^ were assigned to amide I and amide II vibrations, respectively, suggesting the involvement of proteinaceous components. The band at 1231 cm^−1^ may be related to amide III, C–N stretching, or phosphate-associated vibrations, while the band at 1055 cm^−1^ was mainly associated with C–O and C–O–C stretching vibrations in polysaccharides or glycoproteins [13].

Compared with selenium-free JD-21 samples, selenium-enriched JD-21 samples showed stronger amide- and carbohydrate-related absorption bands. These results suggest that protein- and polysaccharide-like biomolecules may be associated with JD-21-derived SeNPs and may act as potential capping or stabilizing agents. However, FT-IR analysis alone cannot definitively determine the complete surface chemical structure of SeNPs, and further surface-specific analyses would be required for more detailed characterization.

### 3.5. Beneficial Properties of JD-21

**Figure 5:**
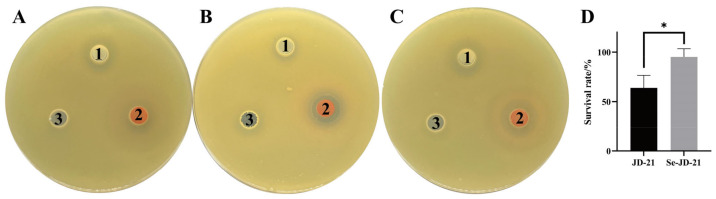
Antibacterial activity of JD-21 before and after selenium enrichment and survival under simulated gastric conditions. (**A**–**C**) Inhibition of three foodborne pathogens by culture supernatants of JD-21 and selenium-enriched JD-21 (Se-JD-21): (**A**) *Escherichia coli*, (**B**) *Staphylococcus aureus* and (**C**) *Salmonella enteritidis*. The numbered wells indicate different treatments: 1, cell-free culture supernatant of JD-21; 2, cell-free culture supernatant of JD-21 cultivated with 5 mmol/L Na_2_SeO_3_, namely Se-JD-21; 3, sterile MRS medium as the negative control. The orange-red color in well 2 was due to selenium enrichment during JD-21 cultivation. The inhibition zone diameters are presented as mean ± SD (*n* = 3): *E. coli*, 14.75 ± 0.35 mm for JD-21 and 32.15 ± 0.21 mm for Se-JD-21; *S. aureus*, 10.67 ± 0.29 mm for JD-21 and 18.90 ± 0.17 mm for Se-JD-21; *S. enteritidis*, 16.87 ± 0.23 mm for JD-21 and 29.83 ± 0.29 mm for Se-JD-21. (**D**) Survival of JD-21 and Se-JD-21 after 3 h of incubation in simulated gastric juice. Differences were considered statistically significant at *p* < 0.05. Single asterisk (*) indicates significant difference between the two groups (*p* < 0.05).

To evaluate the antibacterial properties of JD-21 before and after selenium enrichment, three typical foodborne pathogenic bacteria were selected as indicator strains. The inhibition zones produced by Se-enriched JD-21 (Se-JD-21) and non-enriched JD-21 cultures against *Escherichia coli* were 32.15 ± 0.21 mm and 14.75 ± 0.35 mm, respectively. Against *Staphylococcus aureus*, the inhibition zones were 18.90 ± 0.17 mm for Se-JD-21 and 10.67 ± 0.29 mm for JD-21. For *Salmonella enteritidis*, Se-JD-21 and JD-21 produced inhibition zones of 29.83 ± 0.29 mm and 16.87 ± 0.23 mm, respectively. These results indicate that JD-21 exhibits inhibitory activity against all three pathogens and that selenium enrichment markedly enhances its antibacterial effect (Figure 5A–C). Consistently, Yang et al. [33] reported that selenium-enriched *Lactobacillus delbrueckii* ssp. *bulgaricus* (XN-L-Se) produced an inhibition zone of 18.05 ± 0.06 mm against *Staph. aureus* XN-SA and that 12 µg/mL sodium selenite alone exhibited strong antibacterial activity, with an inhibition zone of 33.01 ± 0.22 mm. Together, these findings support the notion that selenium enrichment can enhance the antibacterial capacity of lactic acid bacteria.

The tolerance of JD-21 and Se-JD-21 to simulated gastric conditions was further assessed. After 3 h of exposure to artificial gastric juice, the survival rate of JD-21 was 61.54 ± 12.73%, whereas Se-JD-21 showed a significantly higher survival rate of 92.86 ± 8.25% (Figure 5D). Lv et al. [34] reported that sixteen lactic acid bacteria strains displayed survival rates of 50.95–73.23% in simulated gastric fluid and 40.01–52.98% in simulated intestinal fluid. In our study, both JD-21 and Se-JD-21 exhibited survival rates above 60% in simulated gastric juice, and selenium enrichment further improved acid tolerance. These results suggest that JD-21 possesses good resistance to gastric acidity and has promising potential as an intestinal probiotic candidate.

### 3.6. Anti-Infective Activity of SeNPs Against Streptococcus suis

To assess the health-related potential of selenium nanoparticles (SeNPs) in maintaining nutritional status and antioxidant balance during physiological stress, we evaluated growth performance (body weight and feed intake), systemic and tissue antioxidant activities (GSH-Px, SOD, and T-AOC in serum, liver, and spleen), as well as liver and spleen histomorphology (H&E staining) across the experimental groups.

**Figure 6:**
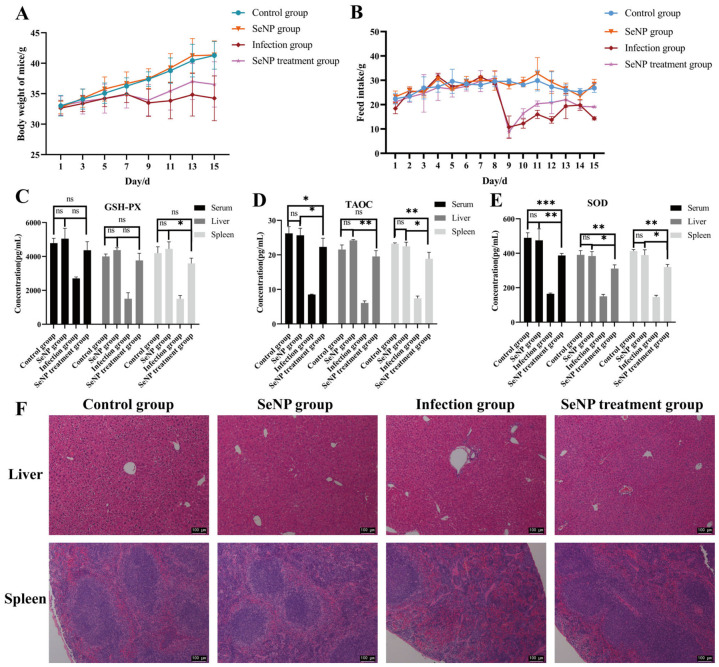
SeNPs mitigate *Streptococcus suis*-induced growth suppression, antioxidant impairment, and liver/spleen injury in mice. (**A**) Body weight changes over the experimental period. (**B**) Feed intake over the experimental period. Mice were assigned to four groups: control, SeNPs, infection (200 μL of *Streptococcus suis*, 1.2 × 10^9^ CFU, injected on day 8), and SeNP treatment (oral-gavage SeNPs plus infection on day 8). SeNPs were administered by oral gavage for 15 consecutive days where applicable. (**C**–**E**) Antioxidant indices measured at sacrifice in serum, liver, and spleen: GSH-Px (**C**), total antioxidant capacity (T-AOC) (**D**), and SOD (**E**), Asterisks indicate significant difference between the groups. (**F**) Representative H&E-stained sections of liver (top) and spleen (bottom) from each group. Data are presented as mean ± SD (*n* = 8 per group). “ns” indicates no significant difference; comparisons without “ns” are significant (*p* < 0.05). Scale bars are shown in the images.

Mice in the control group exhibited a progressive increase in body weight accompanied by a relatively stable pattern of feed intake over the experimental period (Figure 6A), reflecting normal growth and nutritional status. The SeNP group showed comparable trajectories, supporting that the gavaged SeNP regimen was well tolerated and did not induce overt physiological disturbance. Following the day 8 challenge, the infection group displayed a pronounced deterioration in feeding behavior, with a sharp decline in feed intake and a concomitant blunting of body weight gain (Figure 6B), consistent with a stress-associated catabolic state that compromises nutrient intake and growth performance. Importantly, SeNP treatment alleviated these adverse changes, as indicated by a less severe reduction in feed intake and a better maintenance of body weight gain relative to the infection group. From a food-and-nutrition perspective, these outcomes suggest that SeNPs help sustain nutritional resilience—i.e., the capacity to maintain appetite and growth—under conditions that otherwise disrupt feeding and energy balance.

Biochemical indices further supported an SeNP-associated improvement in antioxidant competence. The infection group showed depressed antioxidant enzyme activities and total antioxidant capacity across serum and metabolically active organs, with lower GSH-Px and SOD activities and reduced T-AOC compared with the non-challenged controls (Figure 6C–E). In contrast, SeNP treatment significantly restored these redox-related indices in multiple compartments relative to the infection group (comparisons without “ns” indicating significance), indicating a reinforced enzymatic and non-enzymatic antioxidant network. This pattern is nutritionally meaningful because selenium is a key micronutrient linked to redox homeostasis, and GSH-Px—one of the most representative selenium-dependent antioxidant enzymes—plays a central role in detoxifying peroxides using glutathione as the reducing substrate. The increase in GSH-Px, together with the improvement in SOD and T-AOC, suggests that SeNP administration supports coordinated antioxidant defenses, which may translate into a lower oxidative burden and improved maintenance of physiological functions during stress [5].

Histopathological observations provided qualitative morphological evidence consistent with the physiological and biochemical readouts (Figure 6F). Representative liver sections from the control and SeNP groups displayed preserved hepatic architecture, with relatively regular hepatic cords and sinusoidal spaces and no obvious widespread structural disruption. In the infection group, liver sections displayed visible pathological alterations, including increased structural disorganization and areas suggestive of inflammatory infiltration and/or vascular congestion, indicative of hepatic stress and impaired tissue homeostasis. In the SeNP treatment group, the pathological alterations appeared less pronounced than those in the infection group, with relatively better preservation of the overall hepatic architecture and fewer severe disruptive features, which was consistent with the improvement in antioxidant-related indicators. Similarly, spleen sections from the control and SeNP groups showed an intact overall organization, whereas the infection group exhibited visible splenic alterations, including disturbed splenic architecture and more prominent red-pulp-associated changes, suggesting immune-related tissue perturbation. These changes appeared milder in the SeNP treatment group than in the infection group, with relatively better preservation of splenic structural integrity. Collectively, these representative H&E observations suggest that JD-21-derived SeNPs may help alleviate infection-associated liver and spleen tissue injury.

### 3.7. Transcriptomic Analysis

**Figure 7:**
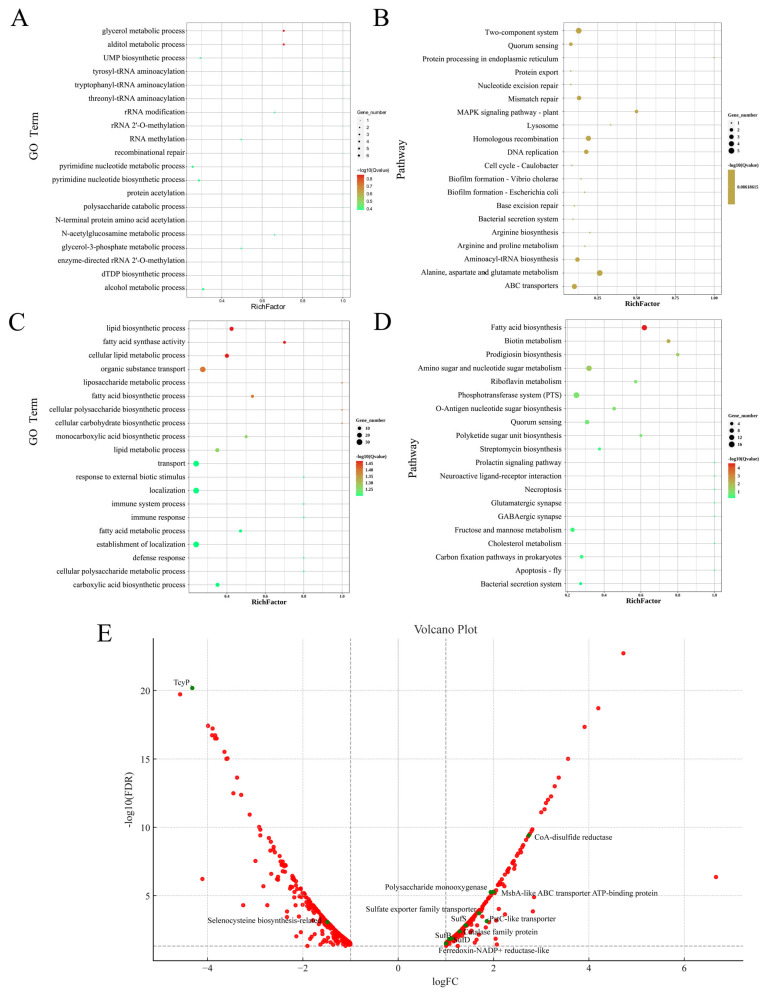
Transcriptomic responses of *Pediococcus acidilactici* JD-21 to Se(IV) stress. (**A**) GO enrichment analysis of up-regulated differentially expressed genes (DEGs) in Se-JD-21 compared with untreated JD-21. (**B**) KEGG enrichment analysis of up-regulated DEGs. (**C**) GO enrichment analysis of down-regulated DEGs. (**D**) KEGG enrichment analysis of down-regulated DEGs. Bubble plots show significantly enriched GO Biological Process terms or KEGG pathways. The *x*-axis represents the RichFactor, calculated as the ratio of DEGs mapped to a given term or pathway to the total number of genes annotated in that term or pathway. The *y*-axis lists enriched terms or pathways. Bubble size represents the number of mapped DEGs, and bubble color indicates enrichment significance as −log_10_(Q-value). (**E**) Volcano plot of DEGs in Se-JD-21 compared with untreated JD-21 under 5 mmol/L Na_2_SeO_3_ treatment. The *x*-axis represents log_2_(fold change), and the *y*-axis represents −log_10_(FDR). DEGs were identified using the criteria of FDR < 0.05 and |log_2_FC| > 1. The vertical dashed lines indicate |log_2_FC| = 1, and the horizontal dashed line indicates FDR = 0.05. Red dots represent significantly differentially expressed genes, and green dots highlight 12 selenium-responsive genes: *sufS*, *sufB*, *sufD*, *tcyP*, sulfate exporter, CoA-disulfide reductase, ferredoxin-NADP^+^ reductase-like, catalase, MsbA-like ABC transporter, PstC-like phosphate ABC permease, polysaccharide monooxygenase, and selenocysteine biosynthesis-related factor.

To elucidate how *Pediococcus acidilactici* JD-21 responds to Se(IV) and supports SeNP biogenesis, we compared the transcriptomes of selenite-treated cells (Se-JD-21, 5 mmol/L Na_2_SeO_3_) and untreated controls (JD-21). Under 5 mmol/L selenite, JD-21 exhibited 537 differentially expressed genes (DEGs) relative to the control, including 240 up-regulated and 297 down-regulated genes, indicating extensive transcriptional remodeling under Se(IV) stress (Figure S1). The RNA-seq data generated in this study have been deposited in the NCBI Sequence Read Archive (SRA) under BioProject accession number PRJNA1372184. To further visualize the expression patterns of the most responsive genes, a heatmap was generated using the top 50 DEGs ranked by FDR and absolute log_2_ fold change. The heatmap shows a clear clustering of biological replicates within each group and a distinct separation between JD-21 and Se-JD-21 samples, indicating a robust transcriptional response of JD-21 to Se(IV) exposure (Figure S3). GO and KEGG enrichment analyses showed that up-regulated genes were mainly associated with lipid and fatty acid metabolism, including “lipid biosynthetic/metabolic process”, “fatty acid biosynthetic process”, “fatty-acid synthase activity”, and the KEGG pathway “fatty acid biosynthesis”. In parallel, pathways related to cofactor and vitamin metabolism (such as biotin and riboflavin metabolism) and carbohydrate uptake and utilization were enriched, including amino sugar and nucleotide sugar metabolism, fructose and mannose metabolism, and the phosphotransferase system (PTS) (Figure 7A). Consistent GO terms for cellular polysaccharide/carbohydrate biosynthesis and organic-substance transport were also significantly enriched (Figure 7B). These transcriptional changes resemble those reported in other selenite-reducing bacteria, in which activation of the pentose phosphate pathway (PPP), central carbon metabolism and respiratory/oxidative phosphorylation modules provides NAD(P)H and electron flux required for Se(IV) → Se(0) reduction and SeNP production [8,32,35]. In JD-21, the concomitant up-regulation of membrane lipid and extracellular polymeric substances (EPS)-related genes likely supports both the supply of reducing power and the formation of a polysaccharide-protein matrix that facilitates SeNP nucleation, immobilization and capping, in line with reports that bacterial EPSs can stabilize SeNPs and modulate their bioactivity [11,36].

In contrast, down-regulated genes were enriched in processes related to nucleic acid and ribosome modification and replication/maintenance, including rRNA modification/methylation, multiple aminoacyl-tRNA ligase functions, and pyrimidine/UMP/dTDP biosynthesis, as well as KEGG pathways such as “DNA replication”, “mismatch/base-excision/nucleotide-excision repair” and “homologous recombination” (Figure 7C). Additional down-regulated categories involved subsets of amino acid and nitrogen metabolism and transport/secretion functions, including ABC transporters, bacterial secretion systems, two-component systems and quorum sensing (Figure 7D). Overall, these patterns suggest that JD-21 partially reallocates resources from rapid growth and replication to stress adaptation and detoxification, downshifting energy-intensive processes and selected transport routes while directing reducing equivalents toward Se(IV) reduction and nano-Se biogenesis. This interpretation is consistent with our experimental observations that selenium-enriched JD-21 cells accumulated large amounts of nano-Se and that abundant SeNPs were present both inside and on the surface of the cells, as confirmed by speciation analysis and electron microscopy.

The transcriptomic data also provide clues to transport and redox systems potentially involved in Se(IV) handling. In many bacteria, phosphate and selenite can compete for uptake via low-affinity phosphate transporters such as PitA, so phosphate transport status can influence Se(IV) entry and intracellular fate [37]. More broadly, ABC, RND and MFS transporters are widely used to export toxic compounds and to remodel membrane traffic under stress and have been implicated in metal (loid) tolerance in diverse bacteria [38,39,40]. On the redox side, the SUF Fe–S cluster assembly system is known to function as an oxidative stress and iron-limitation pathway that maintains Fe–S-dependent biochemistry when prosthetic groups are threatened—a scenario compatible with Se(IV) exposure [41,42]. Catalase-mediated H_2_O_2_ scavenging is a canonical antioxidant response that helps preserve intracellular redox balance by converting H_2_O_2_ to water and O_2_ [43]. Although direct evidence linking catalase up-regulation to enhanced Se(IV) clearance and SeNP formation remains limited, transcriptomic studies in selenite-tolerant bacteria show coordinated induction of antioxidant and oxidoreductase genes under Se stress, supporting the view that reinforced catalase/oxidoreductase activity can help maintain redox balance and thereby favor efficient Se(IV) reduction and nanoparticle formation [32,35,44]. Finally, several food-grade lactic acid bacteria have been reported to efficiently bioreduce selenite to SeNPs while maintaining high viability [8,45], underscoring the translational relevance of JD-21’s LAB-like response program under Se(IV). Taken together, the JD-21 transcriptome supports a model in which enhanced reducing power generation, envelope/EPS remodeling and selective transport control are coupled to drive biological selenite reduction and controlled nano-Se deposition.

### 3.8. Key Genes Involved in Selenium-Stress Responses

Based on differential expression and functional relevance to selenium metabolism and tolerance, we selected twelve Se-responsive genes for focused discussion (prioritizing |log_2_FC| > 1 and/or FDR < 0.05; Figure 7E). Collectively, these genes support a coordinated response to Se(IV) challenge in JD-21, featuring reinforced redox buffering, maintenance of Fe-S homeostasis, selective oxyanion transport, and envelope/EPS remodeling that favors SeNP formation (Figure 7E).

Transport-related DEGs further indicate that JD-21 modulates selenium flux at the membrane level. A sulfate-exporter family transporter and a PstC-like phosphate ABC permease were responsive to Se(IV), aligning with evidence that oxyanion transport systems gate selenite entry and speciation. Given the known competition between phosphate and selenite for low-affinity phosphate transport routes (e.g., PitA), the remodeling of phosphate/anion transporters is a plausible strategy to tune intracellular Se(IV) burden and downstream fate [37,46,47]. An MsbA-like ABC transporter (lipid flippase/exporter) was also induced, highlighting the stress-linked reprogramming of envelope lipid trafficking to preserve membrane integrity and/or facilitate efflux of toxic lipid derivatives during Se(IV) exposure [48,49,50].

Redox-supporting enzymes were another prominent feature. Coenzyme A-disulfide reductase (CoADR) and a ferredoxin-NADP^+^ reductase (FNR-like) component were up-regulated, implying reinforcement of NADPH/ferredoxin cycling and low-molecular-weight thiol redox couples. Such changes would help maintain a “reducing budget” for Se(IV) detoxification and SeNP biogenesis [51,52,53]. Consistently, catalase showed an increasing trend, supporting the concept that JD-21 activates antioxidant defenses to limit Se(IV)-elicited ROS and protect intracellular redox balance [54].

Finally, genes linked to polysaccharide remodeling and selenium utilization suggest structural and biochemical support for nanoparticle formation. A polysaccharide monooxygenase (PMO-like) was induced, indicating active cell-wall/EPS remodeling under Se stress. Bacterial EPSs are widely reported to serve as a capping/stabilizing matrix for biogenic SeNPs, consistent with our FT-IR evidence of protein–polysaccharide coatings and the observed extracellular accumulation of SeNPs [3]. Moreover, a selenocysteine biosynthesis-related factor was responsive to Se(IV), implying potential engagement of Sec-machinery-associated selenium utilization/detoxification routes in JD-21.

**Figure 8:**
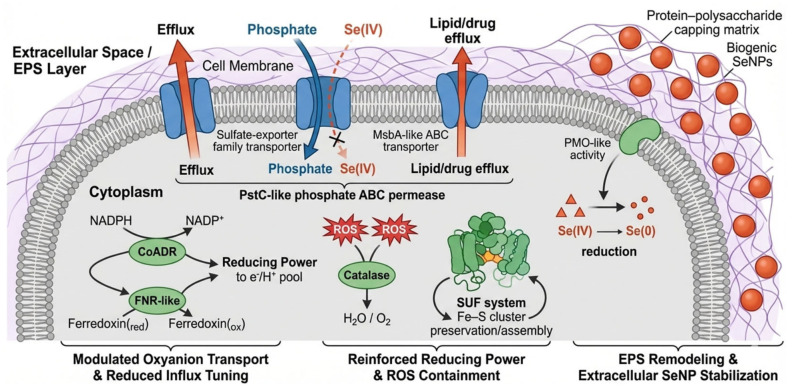
Schematic representation of the integrated molecular mechanism underlying Se(IV) stress response and biogenic selenium nanoparticle (SeNP) formation in strain JD-21. The diagram illustrates a cross-sectional view of the bacterial cell, highlighting four coordinated modules: (i) reinforcement of reducing power and preservation of Fe-S cluster homeostasis via the SUF system, CoADR, and FNR-like components; (ii) activation of antioxidant defenses (e.g., catalase) to contain Se(IV)-induced ROS and maintain intracellular redox balance; (iii) modulation of selenium/oxyanion flux at the membrane level through a sulfate-exporter family transporter, PstC-like phosphate ABC permease, and MsbA-like ABC transporter (lipid flippase/exporter) to tune Se(IV) influx, efflux, and downstream fate; and (iv) envelope and extracellular polymeric substance (EPS) remodeling mediated by a polysaccharide monooxygenase (PMO-like), providing a protein–polysaccharide capping matrix that facilitates nucleation, stabilization, and extracellular accumulation of SeNPs (depicted as orange-red spheres). This multi-layered adaptation program enables efficient Se(IV) detoxification and tolerance in JD-21, consistent with stress response patterns in selenium-reducing lactic acid bacterium-like strains.

Taken together, these gene-level responses outline a coherent mechanism for JD-21 under Se(IV) stress: (i) preservation of Fe-S clusters and expansion of reducing power (SUF system, CoADR, and FNR-like); (ii) antioxidant containment of ROS (catalase); (iii) transporter-level modulation of selenium/oxyanion influx and efflux (sulfate-exporter family, phosphate transporters, and MsbA-like ABC); and (iv) envelope/EPS remodeling that provides a physical matrix for protein–polysaccharide-capped SeNPs. This integrated program is consistent with selenium-reducing bacteria reported previously while reflecting a LAB-type stress adaptation mode in JD-21 (Figure 8).

## 4. Discussion

Microbial reduction of selenite represents an important detoxification strategy and provides a mild biological route for producing elemental selenium nanoparticles (SeNPs). In this study, *Pediococcus acidilactici* JD-21 showed strong Se(IV) tolerance and efficiently converted sodium selenite into red elemental SeNPs, indicating that this strain possesses an effective selenium-stress adaptation system. Compared with many reported lactic acid bacteria, which often tolerate and reduce selenite only at relatively low concentrations, JD-21 showed a stronger ability to survive under Se(IV) stress and accumulate selenium [3]. Selenium speciation, subcellular fractionation and TEM observations collectively suggest that JD-21 preferentially channels Se(IV) toward elemental selenium formation rather than soluble organic selenium accumulation. It should also be noted that SeMet was not detected under the present analytical conditions, rather than being definitively absent from the cells. Previous studies have reported that different LAB strains differ markedly in their selenium biotransformation patterns, with some strains producing SeCys and/or SeMet, whereas others mainly accumulate elemental selenium or selenium nanoparticles [23]. These discrepancies may be related to strain-specific selenium metabolism, selenite concentration, exposure duration, growth phase, and the extraction and speciation methods used. Thus, the selenium metabolism of JD-21 appears to be characterized by efficient Se(IV) reduction and SeNP formation, while its incorporation into detectable selenoamino acids is limited. The visible Se(IV)-reducing activity mainly associated with the cytoplasmic fraction suggests that cytoplasm-associated reductive reactions may play an important role in JD-21-mediated SeNP formation. Meanwhile, electron microscopy showed SeNPs in cell-associated regions and on or near the cell surface, indicating that the resulting nanoparticles may become associated with cellular structures after Se(IV) reduction and nucleation [32,35]. However, the precise spatial process of SeNP formation, intracellular accumulation, and possible release remains to be further verified.

The characterization of JD-21-derived SeNPs further highlights the features of microbial synthesis. TEM analysis showed that the particles were predominantly spherical with an average diameter of approximately 46.4 ± 7.7 nm, a size range generally associated with favorable biological activity. FT-IR analysis indicated that the SeNPs were associated with protein- and polysaccharide-like biomolecules, indicating that bacterial biomolecules may participate in nanoparticle capping and stabilization. Such biomolecular associations are commonly reported for biogenic SeNPs and may contribute to improved colloidal stability, reduced aggregation, and biological compatibility [11,55]. Therefore, the relatively uniform particle morphology and functional performance observed in this study may be partly related to the biomolecular matrix derived from JD-21 cultures, although further surface-specific analyses are needed to clarify the detailed surface chemistry of these SeNPs. Functionally, selenium enrichment markedly strengthened the antibacterial activity of JD-21 against *Escherichia coli, Staphylococcus aureus* and *Salmonella enteritidis* and improved its survival in simulated gastric juice. These effects may result from the combined contribution of SeNPs, selenium-induced metabolic changes and bioactive components produced by JD-21. Similar enhancement of antimicrobial activity after selenium enrichment has been reported in other lactic acid bacteria, supporting the idea that selenium biotransformation can improve the functional performance of probiotic strains [33]. In addition, the improved gastric survival of Se-JD-21 suggests that selenium enrichment may enhance stress resistance, which is important for probiotic application in food and gastrointestinal environments [56].

The in vivo results further suggest that JD-21-derived SeNPs have potential host-protective effects during *Streptococcus suis* infection. In the *Streptococcus suis* infection model, SeNP administration alleviated infection-associated weight loss and feed-intake reduction; partially restored antioxidant-related indicators, including T-AOC, SOD, and GSH-Px activities; and was associated with milder representative histopathological alterations in the liver and spleen. These findings are consistent with the well-known role of selenium in antioxidant defense and redox homeostasis [57]. Similar biochemical effects of SeNPs have been observed across different biological models, including microbial stress adaptation, plant growth responses [58], and mammalian oxidative injury models, suggesting that SeNP activity is closely related to biological context, dose, and redox-associated stress responses. During bacterial infection, excessive oxidative stress and inflammatory responses can impair antioxidant defense systems and contribute to tissue injury. Therefore, JD-21-derived SeNPs may help improve host resistance by enhancing antioxidant capacity, maintaining redox balance, and reducing infection-associated oxidative tissue damage. However, this result should be interpreted as preliminary functional evidence rather than a complete mechanism of host protection. Further studies involving inflammatory cytokines, oxidative stress markers, tissue selenium distribution, dose–response experiments, and quantitative histopathological scoring are needed to clarify the protective mechanism of JD-21-derived SeNPs.

Transcriptomic analysis provides a mechanistic explanation for the Se(IV)-tolerant and SeNP-forming phenotype of JD-21. Phenotypically, JD-21 tolerated 5 mmol/L Na_2_SeO_3_, efficiently reduced Se(IV), accumulated high levels of selenium in biomass, and formed abundant cell-associated SeNPs, while selenium speciation analysis showed that only a small fraction of selenium was detected as soluble organoselenium species. These results suggest that JD-21 mainly copes with high Se(IV) stress through detoxification into elemental selenium rather than extensive incorporation into soluble organic selenium forms. Consistently, RNA-seq showed that genes involved in redox regulation, Fe–S cluster maintenance, cofactor metabolism, cystine/cysteine transport, and antioxidant defense were responsive to Se(IV) exposure. The induction of SUF system genes, CoADR, FNR-like components, catalase, and thiol-related transport systems may help maintain intracellular redox homeostasis [41,42] and provide reducing equivalents for Se(IV) conversion into Se(0), which is also consistent with the strong Se(IV)-reducing activity observed in the cytoplasmic fraction.

In addition, transcriptional changes in phosphate/oxyanion transporters, ABC-type transporters, and MsbA-like membrane transport components suggest that JD-21 may regulate selenium/oxyanion flux and membrane stress during Se(IV) exposure [37]. The up-regulation of carbohydrate utilization, EPS biosynthesis, lipid metabolism, and cell-envelope-related genes further provides a molecular basis for the TEM and FT-IR results, in which nanoscale SeNPs were observed in cell-associated regions and protein-/polysaccharide-related biomolecules were associated with JD-21-derived SeNPs. Together, these findings support an integrated adaptive model in which JD-21 coordinates redox buffering, Se(IV) reduction, transporter remodeling, and EPS/cell-envelope-associated stabilization to promote SeNP formation under selenite stress. However, these transcriptomic results should be regarded as mechanistic clues, and further validation using qRT-PCR, enzyme activity assays, selenium flux analysis, and targeted gene-function studies is needed.

Several *Pediococcus acidilactici* strains with selenium enrichment or selenium-converting abilities have been reported previously. For example, *P. acidilactici* MRS-7 was mainly characterized as a selenium-enriched probiotic strain with organic selenium accumulation and protective effects against patulin-induced jejunal injury [5]. *P. acidilactici* DSM20284 was reported to efficiently reduce selenite and produce spherical SeNPs with protein- and polysaccharide-related capping components [29], while *P. acidilactici* JW-015 was recently developed as a SeNP-enriched probiotic strain with potential anti-hangover effects [59]. These studies demonstrate that selenium transformation is not unique to JD-21 within the species *P. acidilactici*. Compared with these reported strains, JD-21 is distinguished by the combined characterization of strong Se(IV) tolerance, efficient Se(IV) reduction under 5 mmol/L Na_2_SeO_3_, formation of relatively small spherical SeNPs with an average diameter of 46.4 ± 7.7 nm, and functional evaluation in both in vitro and in vivo models. In particular, the present study further integrates selenium speciation, subcellular Se(IV)-reducing activity, TEM/FT-IR characterization, *Streptococcus suis* infection-associated host protection, and RNA-seq-based mechanistic interpretation. Therefore, the main contribution of JD-21 lies not in being the first selenium-converting *P. acidilactici* but in providing an integrated phenotype–mechanism framework for understanding how a food-grade LAB strain tolerates Se(IV), forms SeNPs, and exhibits selenium-associated functional potential.

Despite these findings, several limitations should be acknowledged. First, the transcriptomic results identify candidate pathways and genes associated with Se(IV) stress, but their specific functions in SeNP biosynthesis remain to be experimentally validated. Targeted gene knockout, overexpression or enzyme assays are needed to confirm their roles. Second, although SeNPs showed promising anti-infective activity in mice, long-term safety, bioavailability and dose-dependent effects were not fully evaluated. Third, the performance of JD-21-derived SeNPs or selenium-enriched biomass in real food matrices remains unclear. Future studies should therefore focus on mechanism validation, fermentation optimization, product stability, selenium bioavailability and application evaluation in food systems.

## 5. Conclusions

In this study, a highly Se(IV)-tolerant food-grade lactic acid bacterium, *Pediococcus acidilactici* JD-21 (CCTCC M 20241452), was isolated and characterized. JD-21 tolerated up to 100 mmol/L selenite and efficiently reduced Se(IV) under 5 mmol/L Na_2_SeO_3_ treatment. Selenium-enriched JD-21 accumulated high levels of selenium, while selenium speciation analysis indicated that only a small fraction was present as extractable low-molecular-weight organoselenium species, suggesting that a substantial portion of Se(IV) was likely converted into elemental or nanoparticulate selenium. TEM confirmed the formation of spherical cell-associated SeNPs, with an average diameter of 46.4 ± 7.7 nm, and FT-IR suggested the association of protein- and polysaccharide-related biomolecules with these SeNPs. Selenium enrichment was associated with improved functional properties of JD-21 under the tested conditions, including larger inhibition zones against foodborne pathogens and improved survival in simulated gastric juice. In the *Streptococcus suis* infection model, JD-21-derived SeNPs alleviated infection-associated body weight loss and feed-intake reduction, partially restored antioxidant-related indicators, and were associated with milder representative histopathological alterations in liver and spleen tissues. Transcriptomic analysis further suggested that redox regulation, Fe–S cluster maintenance, transporter remodeling, lipid/EPS metabolism, and cell-envelope-associated processes may contribute to Se(IV) tolerance and SeNP formation in JD-21. However, the present study has several limitations. Selenium speciation results represent extractable selenium species under the applied analytical conditions, and the precise distribution of insoluble or nanoparticulate selenium requires further clarification. In addition, RNA-seq-identified candidate genes and the in vivo protective mechanism require further validation through qRT-PCR, enzyme activity assays, selenium flux analysis, dose–response experiments, inflammatory marker detection, and quantitative histopathological scoring. Overall, JD-21 represents a promising food-grade microbial chassis for biogenic SeNP production and selenium-enriched functional applications.

## Data Availability

The transcriptomic (RNA-seq) data generated in this study have been deposited in the NCBI Sequence Read Archive (SRA) under BioProject accession number PRJNA1372184. All other data supporting the findings of this work are available from the corresponding author upon reasonable request.

## Supplementary Materials

The following supporting information can be downloaded at: https://www.mdpi.com/article/10.3390/foods15142440/s1. Table S1. Working parameters of HPLC and ICP-MS. Table S2. Annotation of the top 50 DEGs shown in the heatmap. Figure S1: Integrated overview of the transcriptomic workflow and DEG landscape of *Pediococcus acidilactici* JD-21 under 5 mmol/L selenite. (A) Transcriptomic analysis workflow for JD-21 selenium tolerance mechanism. (B) Differentially expressed genes (DEGs) in JD-21 under 5 mmol/L selenite vs. control: counts of up and down. Figure S2. Particle size distribution of JD-21-derived SeNPs based on electron microscopy images. (A) Size distribution of SeNPs in intact-cell samples. (B) Size distribution of SeNPs in disrupted-cell samples. Figure S3. Heatmap of the top 50 differentially expressed genes between JD-21 and Se-JD-21.
