# Supplementary material for: Biogenic Selenium Nanoparticles from Food-Grade Pediococcus acidilactici JD-21: Selenite Bioreduction, Enhanced Probiotic Traits, and Antioxidant Protection
Source: Foods. 2026 Jul 9;15(14):2440. doi: 10.3390/foods15142440 (PMC13409270; doi:10.3390/foods15142440)
Supplement: Supplementary file 1 [file foods-15-02440-s001.zip › foods-4377984-supplementary.pdf]

Table S1. Working parameters of HPLC and ICP-MS.

Chromatographic conditions	
Column	250 × 4.1 mm anion exchange Hamilton PRP-X100 (10 μm) column
Mobile phase A	Citric acid 20 mmol/L + 1% methanol , pH 4.0
Mobile phase B	Citric acid 20 mmol/L + 2% methanol , pH 6.0
Flow rate	1.0 mL/min
Injection volume	100 μL

ICP-MS parameters	
Peristaltic pump speed	0.1 rps
Nebulizing gas pressure	300 kPa
Spray chamber temperature	2 °C
Plasma gas flow rate	15.0 L/min
Carrier gas flow rate	1.0 L/min
Auxiliary gas flow rate	1.0 L/min
Nebulizer gas flow rate	1.0 L/min

Table S2. Annotation of the top 50 DEGs shown in the heatmap.

Group	JD-21-1	JD-21-2	JD-21-3	Se-JD-21-1	Se-JD-21-2	Se-JD-21-3	Pvalue	FDR	NR_def
CPU05_RS03715	40.54396	36.70136	41.85466	810.8165	695.3492	766.7561	9.95E-27	1.91E-23	protein
CPU05_RS01930	47780.47	45950.48	48380.3	1813.266	1553.531	1773.468	6.98E-24	6.70E-21	symporter family transporter
CPU05_RS04365	240.2214	222.2987	146.4426	4.517279	7.813286	6.116305	3.02E-23	1.93E-20	carrier protein
CPU05_RS03710	25.84529	39.96604	21.4255	403.2865	387.8609	362.6857	4.16E-22	2.00E-19	protein
CPU05_RS04390	559.0094	430.063	376.252	23.791	20.15606	17.81095	9.80E-21	3.76E-18	dehydratase
CPU05_RS09730	131.3683	197.5069	204.9326	1940.508	2082.05	1800.414	1.40E-20	4.48E-18	protein
CPU05_RS04395	692.2134	495.0351	408.9817	29.16485	24.93506	23.0468	2.14E-20	5.87E-18	carboxylase biotin carboxylase subunit
CPU05_RS04410	204.0877	174.308	114.2523	6.112312	10.91454	6.779912	8.19E-20	1.84E-17	reductase FabI
CPU05_RS04405	542.3989	339.2365	332.5168	25.35862	13.97689	21.25653	8.64E-20	1.84E-17	carboxylase carboxyltransferase subunit alpha
CPU05_RS04355	487.2281	371.8927	258.5613	14.11599	26.85577	15.46202	1.69E-19	3.12E-17	dehydratase FabZ
CPU05_RS04375	445.7192	377.7681	287.2904	15.56695	27.27289	14.51553	1.79E-19	3.12E-17	reductase FabG
CPU05_RS04370	859.2446	605.3462	499.338	43.35249	38.67479	30.75531	1.89E-18	3.03E-16	S-malonyltransferase
CPU05_RS04360	517.7869	411.0991	317.815	27.80573	23.75599	23.17793	6.16E-18	9.10E-16	synthase III
CPU05_RS09965	1216.319	1418.31	1778.99	13555.04	10904.17	13292.92	7.14E-18	9.74E-16	kinase

CPU05_RS04400	421.6442	344.561	313.166<br>6	20.59003	25.51692	18.62471	7.63E-18	9.74E-16	protein
CPU05_RS04385	621.186	393.0953	362.536<br>8	24.12609	38.66389	19.05531	8.12E-18	9.74E-16	carboxylase biotin carboxyl carrier protein subunit
CPU05_RS04380	480.4845	345.2451	274.592<br>8	26.93211	32.01024	17.18921	2.05E-16	2.31E-14	synthase II
CPU05_RS09960	732.2529	807.0231	1014.25<br>5	6927.499	5326.015	6807.247	2.17E-16	2.31E-14	succinate-semialdehyde dehydrogenase
CPU05_RS09565	106.9974	123.7122	152.461	933.6035	871.8665	897.1251	9.72E-16	9.82E-14	system mannose/fructose/sorbos e family transporter subunit IID
CPU05_RS01940	116.7666	98.0759	119.249<br>9	7.106403	2.953347	11.29531	3.34E-15	3.21E-13	domain-containing protein
CPU05_RS01945	113.27	99.6239	127.131<br>5	8.888945	7.853957	8.230127	4.59E-15	4.20E-13	starvation/stationary phase protection protein
CPU05_RS04195	52.46678	63.29249	50.3152<br>8	289.7404	475.4098	344.9187	6.25E-15	5.46E-13	protein
CPU05_RS09575	65.00007	87.00381	114.715<br>2	592.468	539.8719	576.7509	1.15E-14	9.59E-13	sugar transporter subunit IIB
CPU05_RS01545	338.6477	311.7665	296.111<br>5	1647.377	2368.535	1850.225	2.07E-14	1.66E-12	deaminase
CPU05_RS03505	70.77485	57.06775	41.9434<br>9	243.4936	421.9972	363.1307	6.21E-14	4.77E-12	domain-containing protein
CPU05_RS09570	148.2764	166.4684	202.034<br>9	998.6	951.1419	1044.539	1.08E-13	7.95E-12	sugar transporter subunit IIC

CPU05_RS02430	47.65077	44.09323	48.95472	6.195278	3.940138	1.551258	1.66E-13	1.18E-11	sugar transporter subunit IIC
CPU05_RS03875	148.1316	174.9894	168.648	16.49741	17.78476	12.87326	1.37E-12	9.39E-11	family protein
CPU05_RS09580	150.9588	180.3754	201.8846	962.5853	837.0643	913.3042	2.11E-12	1.39E-10	protein
CPU05_RS05120	96.829	108.8855	98.59232	8.82965	10.7173	10.08717	2.31E-12	1.48E-10	family transcriptional regulator
CPU05_RS09430	90.09733	81.61912	88.02157	505.732	339.0615	455.5103	3.06E-12	1.90E-10	family protein
CPU05_RS09585	207.9905	216.4301	275.2362	1117.636	1194.137	1126.341	5.08E-12	3.05E-10	sugar transporter
CPU05_RS03500	95.9853	84.78655	86.72466	430.6814	403.5975	454.8933	6.83E-12	3.88E-10	oxidoreductase
CPU05_RS04830	98.22131	99.08278	82.45438	3.798563	13.10393	10.55705	6.87E-12	3.88E-10	family protein
CPU05_RS08910	1107.555	1081.125	1403.855	6448.848	4416.583	6257.89	8.75E-12	4.80E-10	family protein
CPU05_RS06090	332.6593	286.9901	298.79	32.24165	35.5738	33.34217	1.14E-11	6.06E-10	antiporter NhaC, nhaC
CPU05_RS06860	937.7817	799.1653	903.0247	4044.267	3937.181	4251.575	1.62E-11	8.40E-10	RecA
CPU05_RS09535	218.8532	264.1359	298.5311	32.087	26.8695	29.79892	2.26E-11	1.14E-09	mutarotase
CPU05_RS03180	663.6621	473.3727	684.9973	3115.247	2040.806	2933.056	4.07E-11	2.00E-09	dehydratase
CPU05_RS09490	196.4285	191.7898	170.2911	21.65515	21.87392	22.29172	5.65E-11	2.71E-09	domain-containing protein

CPU05_RS09265	93.40303	66.7442	78.97578	337.9992	357.0131	354.0836	5.85E-11	2.74E-09	protein
CPU05_RS03670	36.22377	44.19755	34.6324	4.626028	3.258709	5.555172	9.30E-11	4.25E-09	sugar transporter subunit IIA
CPU05_RS07465	304.6735	313.8654	322.7884	1511.509	1055.353	1442.336	1.01E-10	4.51E-09	DUF2800 domain-containing protein [Lactobacillaceae]
CPU05_RS04625	64.02218	62.83616	58.47382	7.83182	6.457008	6.452512	1.16E-10	5.05E-09	transporter family protein
CPU05_RS07685	8.952554	9.274828	13.59563	47.91091	45.06236	44.8118	1.59E-10	6.76E-09	DNA polymerase
CPU05_RS02345	139.2146	133.9203	132.3858	15.50932	15.21535	17.89019	1.62E-10	6.77E-09	sugar transporter subunit IIB
CPU05_RS00950	1744.229	1785.977	1906.594	7936.871	6850.545	7649.996	2.04E-10	8.31E-09	PepV
CPU05_RS09560	310.2298	306.4938	356.9256	1294.716	1287.009	1372.156	3.03E-10	1.21E-08	family transcriptional regulator
CPU05_RS02310	481.1853	599.6354	618.6699	73.68929	74.0335	71.92008	3.24E-10	1.27E-08	transaminase (isomerizing)
CPU05_RS10140	1434.283	1256.002	1413.739	5733.631	4620.949	5554.874	7.66E-10	2.89E-08	protein

Figure S1 Integrated overview of the transcriptomic workflow and DEG landscape of *Pediococcus acidilactici* JD-21 under 5 mmol/L selenite. (A) Transcriptomic Analysis Workflow for JD-21 Selenium Tolerance Mechanism. Workflow Overview: This flowchart illustrates the sequential steps involved in analyzing the transcriptomic response of JD-21 strain to selenium treatment. It begins with strain cultivation and branches into experimental (selenium-treated) and control groups. The process then converges through RNA extraction, sequencing data generation, and bioinformatics analysis steps, culminating in functional annotation and differential expression analysis. (B) Differentially expressed genes (DEGs) in JD-21 under 5 mmol/L selenite vs control: counts of Up and Down. Bar chart summarizing the numbers of down-regulated and up-regulated genes for the A-vs-B comparison (JD-21, 5 mmol/L Na_2SeO_3 vs control). The y-axis shows the number of DEGs; blue indicates Down (297; 55.3%) and red indicates Up (240; 44.7%). Values and percentages are annotated above each bar. This panel provides an overview of the transcriptional directionality prior to enrichment analyses.

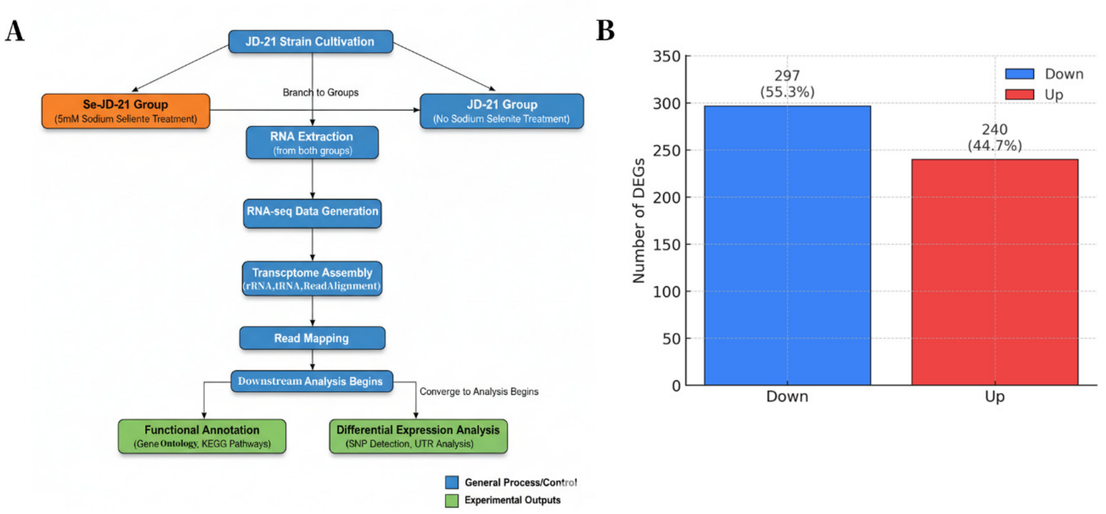

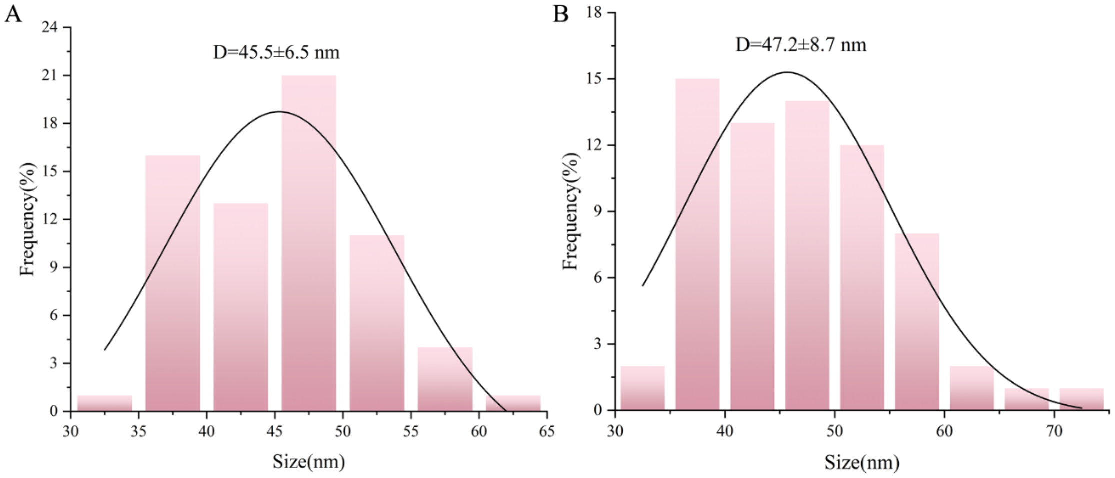

Figure S2. Particle-size distribution of JD-21-derived SeNPs based on electron microscopy images. Particle sizes were measured using Nano Measurer software. (A) Size distribution of SeNPs in intact-cell samples. The average particle diameter was 45.5 ± 6.5 nm (n = 67). (B) Size distribution of SeNPs in disrupted-cell samples. The average particle diameter was 47.2 ± 8.7 nm (n = 68). The combined analysis included 135 clearly distinguishable particles, with an overall average diameter of 46.4 ± 7.7 nm. Data are presented as mean ± SD.

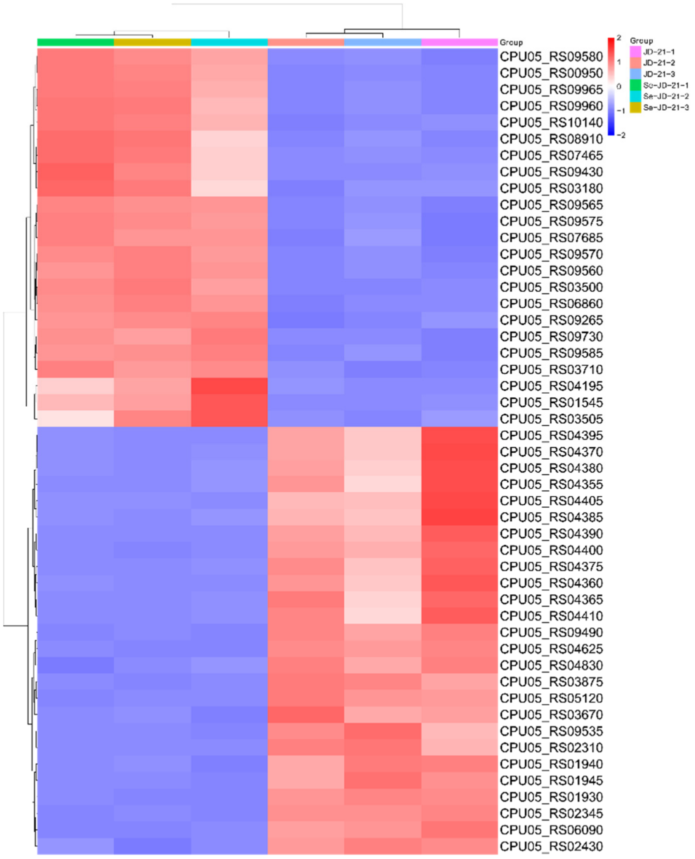

Figure S3. Heatmap of the top 50 differentially expressed genes between JD-21 and Se-JD-21.
The top 50 DEGs were selected according to FDR and absolute log_2 fold change. Rows represent genes and columns represent biological replicates. Expression values were normalized by Z-score across samples. Hierarchical clustering was performed based on normalized expression patterns.
